# Targeted next-generation sequencing-based pathogens detection in children with severe pneumonia in the pediatric intensive care unit

**DOI:** 10.3389/fped.2026.1825458

**Published:** 2026-04-28

**Authors:** Ning Zhu, Yanqing Tang, Zhenjian Zhang, Dingjian Ye, Youjun Xie, Dongming Li, Zhikun Liang

**Affiliations:** 1 Department of Laboratory Medicine, Maternal and Child Health Hospital of Guangxi Zhuang Autonomous Region, Nanning, China; 2 Genetic and Metabolic Central Laboratory, Maternal and Child Health Hospital of Guangxi Zhuang Autonomous Region, Nanning, China; 3 Department of Pediatric Respiratory Medicine, Maternal and Child Health Hospital of Guangxi Zhuang Autonomous Region, Nanning, China; 4 Department of Medical Record Information, Maternal and Child Health Hospital of Guangxi Zhuang Autonomous Region, Nanning, China; 5 Department of Critical Care Medicine, Maternal and Child Health Hospital of Guangxi Zhuang Autonomous Region, Nanning, China

**Keywords:** infection, pathogens, pediatric intensive care unit, severe pneumonia, targeted next-generation sequencing

## Abstract

**Background:**

Severe pneumonia is predominantly caused by multiple pathogenic microorganisms. Conventional microbiological tests (CMTs) often have limited sensitivity or a narrow detection spectrum of pathogens, potentially leading to delayed diagnosis. Based on multiplex PCR and high-throughput sequencing, targeted next-generation sequencing (tNGS) overcomes previous limitations. However, research on the use of tNGS in severe pediatric pneumonia remains quite scarce. This study focused on comparing the effectiveness of tNGS with that of CMTs among children with severe pneumonia in the pediatric intensive care unit (PICU).

**Methods:**

This study enrolled 344 patients with severe pneumonia, admitted to PICU at the Maternal and Child Health Hospital in Guangxi Province, China, between June 2021 and December 2024. Both tNGS and CMTs were performed to detect pathogenic microorganisms in all patients.

**Results:**

Pathogens were identified in 343 out of 344 patients, with the detection rate by tNGS significantly higher than that of CMTs (99.71% vs. 49.71%, *p* < 0.05). Among these cases, 290 (84.30%) involved multiple pathogen infections. Bacterial-viral co-infection was the predominant type (31.20%, 107/343). 86 pathogens were identified, among which the four most prevalent were respiratory syncytial virus (39.24%), cytomegalovirus (28.20%), rhinovirus (24.13%), and Mycoplasma pneumoniae (22.67%). Notably, tNGS detected antimicrobial resistance genes in 10.17% (35/344) of cases, including the A2063G macrolide-resistance mutation in 29.49% (23/78) of Mp and 7.69% (1/13) of Bordetella pertussis isolates. Additionally, 20% (4/20) of Staphylococcus aureus carried the mecA gene, while two bla_CTX-M and five bla_NDM resistance genes were identified among Enterobacteriaceae and non-fermenting Gram-negative bacilli. One patient died during hospitalization. In 35 patients, treatment was withdrawn per family request, and 14 of them died shortly after discharge. The length of hospital stay was 15 days.

**Conclusions:**

This study demonstrates that tNGS substantially enhances etiologic diagnosis in children in the PICU with severe pneumonia. It revealed a high burden of viral and mixed infections and identified resistance genes with potential implications for antimicrobial therapy.

## 1 Introduction

Pulmonary infection is the predominant cause of hospitalization and death among children aged < 5 years worldwide, involving a variety of viral, bacterial, and fungal pathogens (1). Severe pneumonia is a serious condition in children, accounting for nearly 11.5% of all pneumonia-related hospitalizations and approximately 12% of deaths among children admitted to the pediatric intensive care unit (PICU) (2, 3). Importantly, timely and accurate identification of causative pathogens of severe pneumonia, followed by sensitive drug therapy, is essential for improving clinical outcomes (4, 5). The COVID-19 pandemic, alongside empirical antimicrobial therapy, has reshaped the epidemiology of respiratory pathogens and increased the incidence of co-infections (6, 7).

Recent studies indicate that conventional microbiological tests (CMTs), which include multiple laboratory techniques such as targeted PCR assays, serology, and culture, exhibit sensitivities ranging from 65% to 90%, primarily detecting common pathogens (8). These limitations frequently impede timely and accurate diagnosis. Several studies indicate that metagenomic next-generation sequencing (mNGS) has enhanced the identification of pathogens, especially for rare or mixed infections (9–12). Nevertheless, the widespread use of mNGS is hindered by the considerable cost and complex technique. Emerging as a promising detection technology, targeted next-generation sequencing (tNGS) integrates multiplex PCR with high-throughput sequencing. The range of detectable pathogens is slightly narrower than that of mNGS, however, tNGS has distinct advantages regarding the diagnostic turnaround time and cost (11–13). A preliminary report confirmed that tNGS detected respiratory pathogens at approximately one-quarter of the cost of mNGS (13). Furthermore, another study showed comparable diagnostic accuracy between tNGS and mNGS in patients with lower respiratory tract infection (14). However, large-scale clinical evaluations of tNGS in pediatric severe pneumonia remain scarce.

To fill this gap, we conducted a comprehensive analysis utilizing a tNGS platform to analyze samples from 344 children admitted to the PICU, who met the criteria for severe pneumonia. Our aims were to (I) evaluate the diagnostic effectiveness of tNGS with CMTs; (II) investigate the distribution of pathogens and types of infection; and (III) examine the profiles of antimicrobial resistance genes.

## 2 Methods

### 2.1 Study patients

This study retrospectively focused on 344 patients admitted to the PICU, and all were diagnosed with severe pneumonia at a pediatric tertiary center—the Maternal and Child Health Hospital of Guangxi Province between June 2021 and December 2024. The diagnosis of severe pediatric pneumonia met the criteria of 2013 revised guidelines for the management of community-acquired pneumonia in children (15). This study was granted a waiver of written informed consent, and the protocol received approval from the hospital's Ethics Committee.

### 2.2 Sample collection and conventional testing

Sputum and bronchoalveolar lavage fluid (BALF) samples were obtained both at admission and whenever the patient's condition worsened. CMTs included culture, serology, and PCR assays. Indirect immunofluorescence assay (IFA; VIRCELL) targeted nine pathogens: Mycoplasma pneumoniae (Mp), influenza A and B viruses, Chlamydophila pneumoniae, parainfluenza virus, respiratory syncytial virus (RSV), Coxiella burnetii, adenovirus, and Legionella pneumophila. Single-target PCR assays were performed for Epstein–Barr virus, cytomegalovirus, Mp, RSV, adenovirus, and enteroviruses. Multiplex PCR assays targeted Mp, adenovirus, RSV, influenza A/B, and rhinovirus.

### 2.3 Targeted next-generation sequencing

Nucleic acids were extracted using the KS118-BYTQ-24 kit (King Create Co., Ltd, Guangzhou, China). Library preparation employed the KS608-100HXD96 ultra-multiplex PCR system (King Create Co., Ltd) paired with high-throughput sequencing. The assay was designed to detect up to 225 respiratory pathogens (91 bacteria, 81 viruses, 43 fungi, and 10 other species) and 20 antimicrobial resistance loci, including genes associated with resistance to cephalosporins, extended-spectrum β-lactamases, methicillin-resistant Staphylococcus aureus, and mutations in Mp, Bordetella pertussis, and Aspergillus fumigatus (Supplementary Table 1). The detailed methodology has been described previously (16). Sequencing data were independently analyzed by two laboratory physicians, and clinical correlation was performed by two clinicians blinded to each other's assessments.

### 2.4 Statistical methods

Data analyses were performed using IBM SPSS version 25.0. For categorical variables, data are presented as counts and percentages, using the *χ*^2^ (chi-square) test to compare pathogen detection rates between tNGS and CMTs at a 5% significance level. For continuous variables with a non-normal distribution, data are expressed as median and interquartile range (IQR).

## 3 Results

### 3.1 Clinical characteristics

Among 344 pediatric patients with severe pneumonia, 121 were female and 223 were male. The age distribution ranged between 28 days and 14 years; 318 patients were younger than 5 years, and only 26 were older than 5 years. Most patients were referred from other hospitals, followed by direct admissions via outpatient or emergency departments, with a smaller proportion transferred from general pediatric wards (Table 1).

**Table 1:** Clinical characteristics of pediatric patients with severe pneumonia.

Characteristic	Number	Percentage (%)
Gender		
Male	223	64.83
Female	121	35.17
Age		
<1year	176	51.16
1–3years	101	29.36
3–5years	41	11.92
>5years	26	7.56
Specimen Type		
Sputum	63	18.31
Bronchoalveolar lavage fluid	281	81.69
Admission mode		
Outpatient/Emergency	149	43.32
General ward transfer	19	5.52
Referral from other institutions	176	51.16
Comorbidities	145	42.15

Comorbidities were present in 153 cases, including congenital heart disease (*n* = 75), congenital laryngomalacia (*n* = 28), bronchopulmonary dysplasia (*n* = 19), esophagotracheal fistula or esophageal atresia (*n* = 7), spinal muscular atrophy (*n* = 5), post-liver transplantation or under chemotherapy for leukemia (*n* = 5), hydrocephalus (*n* = 3), connective tissue disease (*n* = 2), and nephrotic syndrome (*n* = 1).

### 3.2 Pathogen spectrum

**Figure 1:**
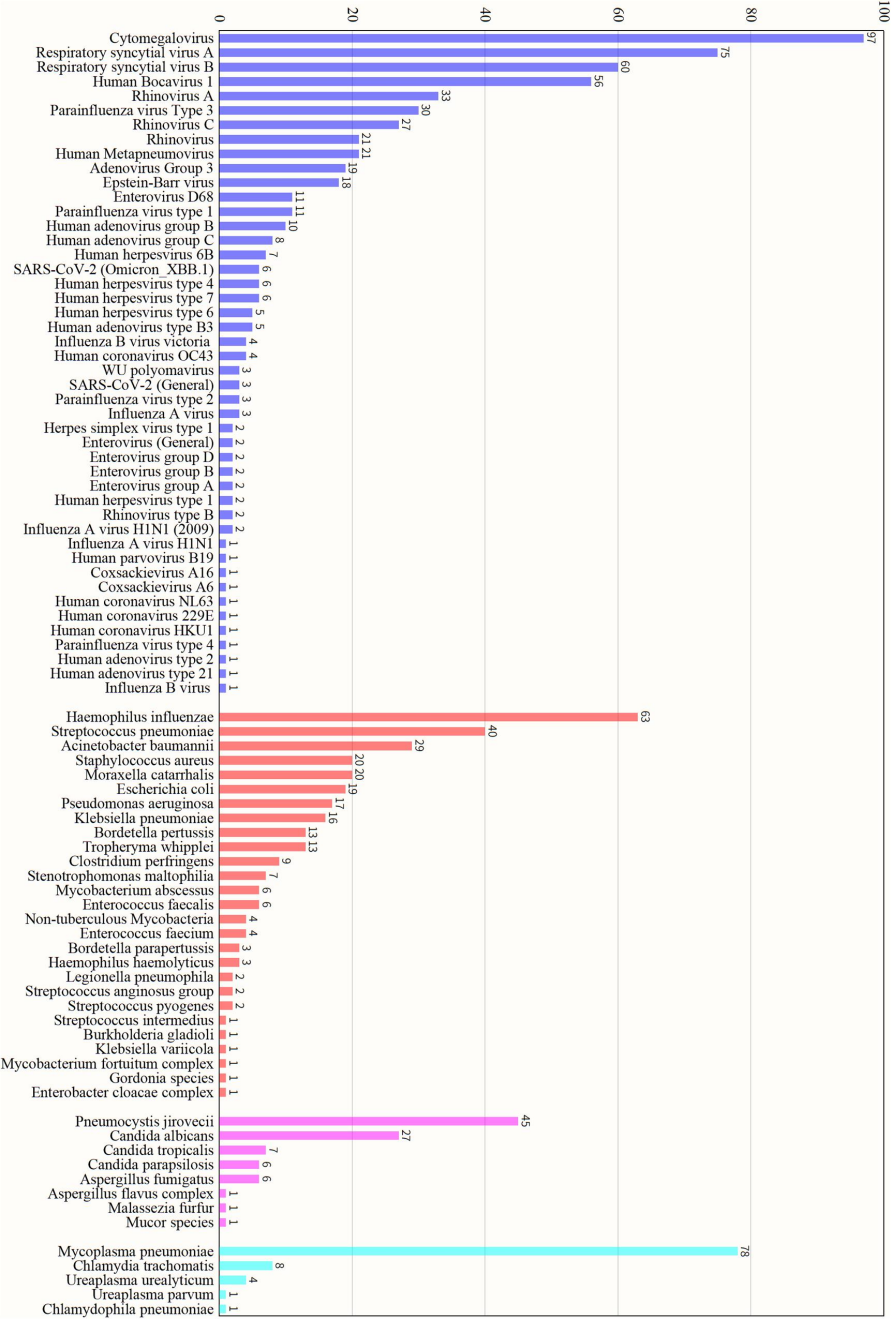
Pathogens distribution by tNGS in PICU patients with severe pneumonia.

Among the 344 patients diagnosed with severe pneumonia, tNGS identified pathogens in 343 cases, achieving a detection rate of 99.71% (Figure 1). A total of 86 pathogens were detected. Among the 86 detected pathogens, viruses accounted for slightly more than half (53.49%, 46/86), with RSV being the most common at 39.24% (135/344). RSV was subclassified into RSV-A (21.80%, 75/344) and RSV-B (17.44%, 60/344), followed by cytomegalovirus (28.20%, 97/344) and rhinovirus (24.13%, 83/344). Bacteria accounted for nearly one-third (31.40%, 27/86), predominantly Haemophilus influenzae (18.31%, 63/344), Streptococcus pneumoniae (11.63%, 40/344), and Acinetobacter baumannii (8.43%, 29/344). Fungi constituted 9.30% (8/86), including Pneumocystis jirovecii (13.08%, 45/344) and Candida albicans (7.85%, 27/344). Mycoplasma and Chlamydia species together made up 5.81% (5/86), and Mp was detected in 22.67% (78/344) of samples.

**Figure 2:**
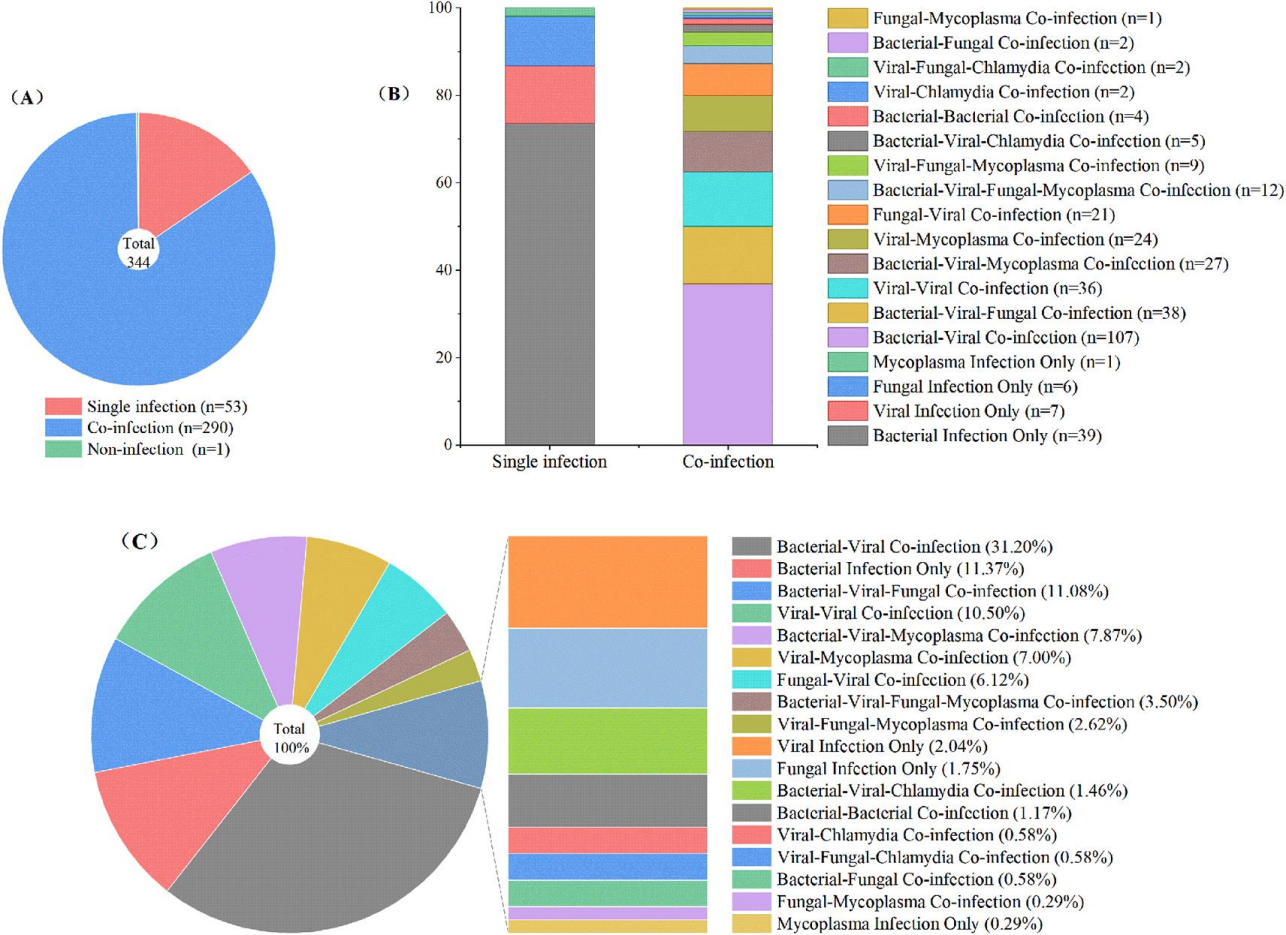
Pathogen profiles in 344 pediatric patients with severe pneumonia. **(A)** Infection status of the cohort; **(B)** composition of single and co-infections; **(C)** distribution of pathogen combination profiles.

Co-infections were frequent (84.30%; Figure 2A), with up to nine pathogens detected in a single specimen. Eighteen distinct infection patterns were identified (Figure 2B), with bacterial-viral co-infections constituting the most prevalent pattern (31.20%), followed by bacterial infections only (11.37%), bacterial-viral-fungal co-infections (11.08%), and viral-viral co-infections (10.50%) (Figure 2C).

### 3.3 Comparative performance of tNGS and CMTs

**Figure 3:**
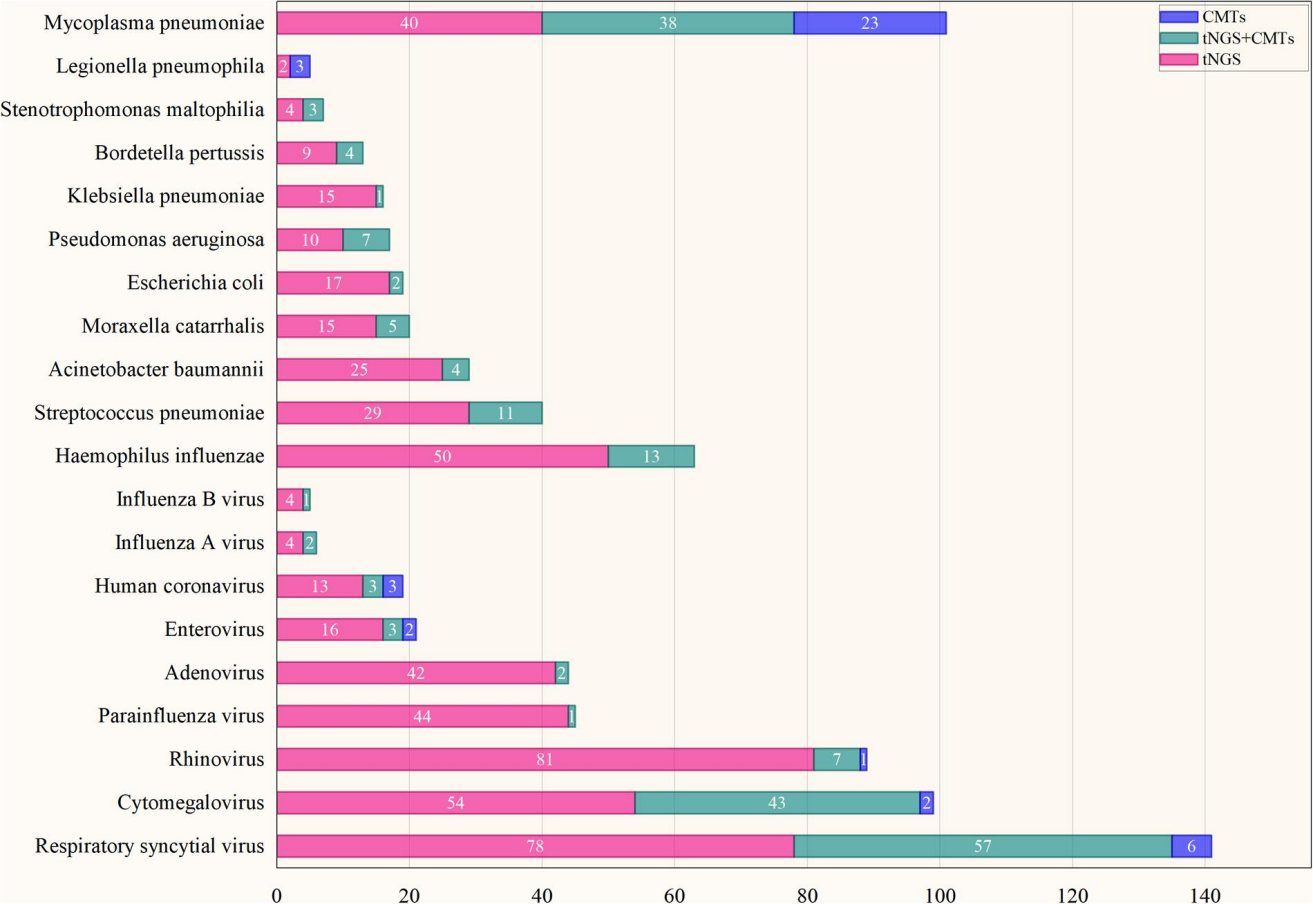
Detection patterns of 20 pathogens by CMTs and tNGS.

CMTs detected pathogens in 49.71% (171/344) of cases, significantly lower than that of tNGS (*p* < 0.05). Complete concordance between CMTs and tNGS was observed in only 4.09% (7/171) of CMTs-positive cases; partial concordance, defined as at least one overlapping pathogen, occurred in 72.52% (124/171); discordance was noted in 23.39% (40/171). CMTs identified merely 20 pathogen species, whereas tNGS detected 86 (Figure 3). PCR assays recognized two enterovirus cases and one rhinovirus case not initially detected by tNGS, but repeat PCR testing of the rhinovirus-positive sample was negative. Serology identified 33 patients positive for 40 pathogens; notably, all corresponding respiratory specimens tested negative by both tNGS and PCR (Figure 3). Among serologically diagnosed Mp cases (*n* = 23), 78.26% (18/23) received targeted antimicrobial therapy.

### 3.4 Detection of antimicrobial resistance genes

A total of 35 cases (10.17% of 344) were found to carry antimicrobial resistance genes detected by tNGS. The macrolide resistance-associated A2063G mutation was identified in 29.49% (23/78) of Mp isolates. Among these patients, erythromycin was administered to one patient, and levofloxacin to two patients. The same mutation was detected in 7.69% (1/13) of Bordetella pertussis isolates. The methicillin resistance gene mecA was present in 20% (4/20) of Staphylococcus aureus isolates. Among Enterobacteriaceae and non-fermenting Gram-negative bacilli, two isolates carried the β-lactamase gene bla_CTX-M, and five carried the carbapenemase gene bla_NDM.

### 3.5 Clinical outcomes and healthcare burden

**Figure 4:**
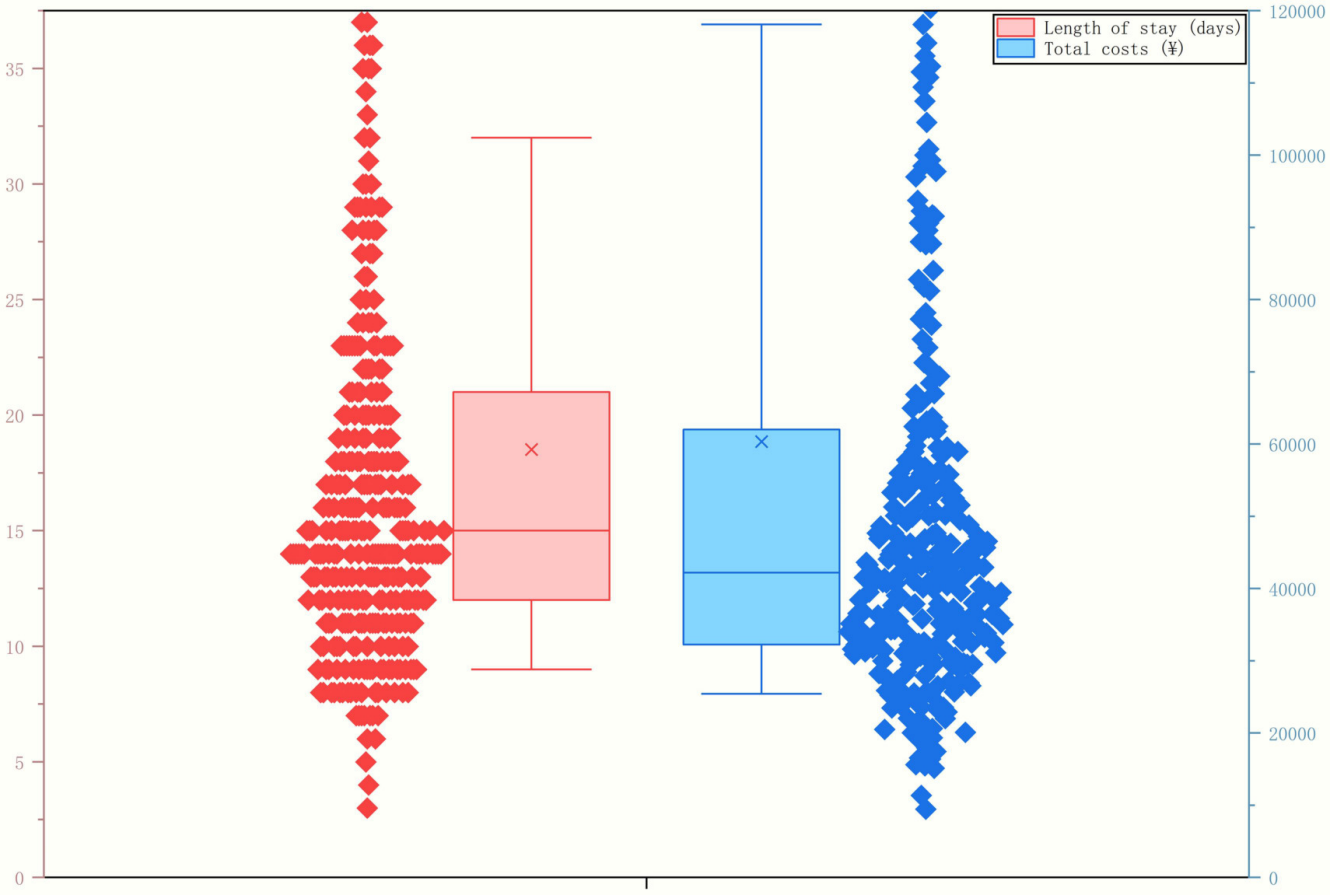
Length of hospital stay and medical costs for pediatric patients with severe pneumonia in PICU.

Of the 344 patients, 202 (58.72%) attained clinical stability and were transferred to general wards, while 100 (29.07%) were discharged directly following clinical improvement. A total of 42 (12.21%) had a poor prognosis. Among these patients, six (1.74%) were transferred to specialized hospitals due to comorbidities involving other specialties. Unfortunately, one patient (0.29%) died in hospital because of multiple organ dysfunction. At the families’ request, 35 patients (10.17%) were discharged early due to an unsatisfactory prognosis, and regrettably, 14 (4.07%) of these patients died shortly after discharge. Children with severe pneumonia had an prolonged hospital stay [median, 15 (IQR, 12-21) days], and hospitalization costs were substantial [median, ¥42,347 (IQR, ¥32,231-¥61,991) (Figure 4)]. Insurance covered approximately 55% of the costs [median, 54.88% (IQR, 48.21%–63.45%)].

## 4 Discussion

In our study, we utilized tNGS to identify pathogens in children admitted to the PICU for severe pneumonia at a pediatric tertiary center in Guangxi. Among 344 BALF and sputum samples, tNGS detected 86 underlying pathogenic microorganisms, achieving a higher positivity rate than CMTs (99.71% vs. 49.71%). Compared with previous reports, the pathogen detection rate was similar (99.71% vs. 99.01%), whereas the diversity of identified species increased significantly (86 vs. 39) (17). Another study reported that mNGS detected 78 pathogens, with a detection rate of 97.61% (3). These findings highlight the remarkable performance of tNGS, indicating that it is a sensitive and comprehensive tool for pathogen identification in patients with severe pneumonia.

Viruses represented the most frequently identified pathogens in pediatric patients with severe pneumonia, constituting 53.49% of the 86 detected species. RSV was the predominant virus detected, followed by cytomegalovirus and rhinovirus. These findings align with earlier reports (3, 4). RSV is attributed to substantial disease burden in children (18). Epidemiological investigations have indicated that respiratory symptoms caused by rhinovirus infections tend to be mild, however, it has also emerged as a prominent cause of severe pneumonia in children (19, 20). In the present study, the elevated detection rates of cytomegalovirus (28.20%) and rhinovirus (24.13%) observed may be explained by the high comorbidity (45.15%), and infants under one year old accounted for 51.16%. It is reported that reactivation of CMV is frequently observed in ICU patients and is closely associated with severe complications (21, 22). Nevertheless, the precise pathogenic role of CMV remains difficult to define.

Co-infections were frequently detected in patients with respiratory infectious, accounting for 57.80% to 76.32% of cases (3, 18). In this study, the prevalence of co-infections was high (84.30%), and the most common pattern was viral and bacterial mixed infections (31.20%). We also found that among single infection cases, 73.58% (39/53) were caused by a single bacterial pathogen. tNGS identified 27 different bacterial species, with Haemophilus influenzae, Streptococcus pneumoniae, and Acinetobacter baumannii appearing most often. Jung et al. found that bacteria in viral-bacterial co-infections are mainly opportunistic pathogens (23). The Global Burden of Disease study 2016 revealed that Streptococcus pneumoniae was the leading cause of global morbidity and mortality due to lower respiratory infection, contributing to over one million deaths in children under five (24). These findings highlight the crucial role of bacterial infections in severe respiratory diseases among children. tNGS detected four key antimicrobial resistance genes—A2063G (macrolide resistance), mecA, bla_CTX-M, and bla_NDM—in 20 isolates. These findings are consistent with the research of Liu and colleagues, who reported that the presence of resistance genes does not always predict actual drug resistance phenotypes (24). Due to the low detection rate of microbial culture, we suggest that profiling resistance genes may provide guidance for empirical antibiotic therapy.

Following the COVID-19 pandemic in late 2023, Mp caused community-acquired pneumonia outbreaks worldwide (25, 26). In China, a widespread outbreak of Mp infections was also observed (27–31). In our study, Mp was detected by tNGS in approximately 23% (78 of 344 patients) of severe pneumonia cases. Nearly all these patients had co-infections with other bacteria and viruses. Yang et al. reported that 37.9% (80/211) of patients with severe Mp pneumonia (SMPP) had a significantly high co-infection in 2012 (32). A systematic review shows a significant increase in the prevalence of macrolide-resistant Mp infections globally (26). Our observation showed that approximately 30% of Mp isolates were found to carry macrolide resistance-associated A2063G mutation, and azithromycin remained the preferred treatment. Only one patient was switched to erythromycin and two to levofloxacin due to a poor prognosis. Chen et al. reported that early use of minocycline might reduce the burden of macrolide-resistant Mp, with side effects occurring in only 0.7% (*n* = 1) of cases (33). This suggests that second-line antibiotics should be used even in patients younger than 2 years old diagnosed with severe MPP (34).

Due to the complexity of intensive care, associated serious complications, and challenges in diagnosis and treatment, data on the medical burden of children with severe pneumonia remain limited (35–37). In our cohort, the median hospital stay was 15 days. The hospitalization costs were substantial, reaching ¥42,347, which accounted for nearly 20% of the annual disposable income (38). At the families’ request, approximately 10% (*n* = 35) of patients were discharged early due to an unsatisfactory prognosis. Of these, 4.07% (*n* = 14) died shortly after discharge. These observations underscore the urgent need to provide stronger financial support for affected families.

Although our study provided insights, it had some limitations. One limitation is that patients were mainly referred from other hospitals, while the retrospective study was conducted at a single center. Another limitation involves common empirical antimicrobial treatments possibly altering the spectrum of certain bacterial pathogens, and the correlation between resistance phenotypes and resistance genes requires further validation. Finally, although tNGS covers extensive pathogen panel, distinguishing infection from colonization and contamination remains challenging.

In conclusion, tNGS shows notable performance in identifying pathogens related to severe pneumonia in PICU. Co-infections were found in 84.3% of cases, most often involving bacterial and viral mixed infections. It also finds important genes linked to antimicrobial resistance. The treatment burden for children with severe pneumonia is substantial. These findings strongly support using tNGS as a routine tool alongside other tests to improve precision therapy and antimicrobial management in the PICU. Further multi-center research should be conducted to verify these findings.

## Data Availability

The raw data supporting the conclusions of this article will be made available by the authors, without undue reservation.

## References

[1] GBD 2021 Lower Respiratory Infections and Antimicrobial Resistance Collaborators. Global, regional, and national incidence and mortality burden of non-COVID-19 lower respiratory infections and aetiologies, 1990–2021: a systematic analysis from the global burden of disease study 2021. Lancet Infect Dis. (2024) 24(9):974–1002. 10.1016/S1473-3099(24)00176-2 38636536 PMC11339187

[2] Rudan I O'Brien KL Nair H Liu L Theodoratou E Qazi S Epidemiology and etiology of childhood pneumonia in 2010: estimates of incidence, severe morbidity, mortality, underlying risk factors and causative pathogens for 192 countries. J Glob Health. (2013) 3(1):010401. 10.7189/jogh.03.010401 23826505 PMC3700032

[3] Li M Wang J Yao Z Liao H Su S Yang X Metagenomic-based pathogen surveillance for children with severe pneumonia in pediatric intensive care unit. Front Public Health. (2023) 11:1177069. 10.3389/fpubh.2023.1177069 37397737 PMC10309210

[4] Qin L Liang M Song J Chen P Zhang S Zhou Y Utilizing targeted next-generation sequencing for rapid, accurate, and cost-effective pathogen detection in lower respiratory tract infections. Infect Drug Resist. (2025) 18:329–40. 10.2147/IDR.S494558 39840396 PMC11748758

[5] Cui S Guo R Chen C Zhang Y Meng J Liu L Next-Generation sequencing for characterizing respiratory tract virome and improving detection of viral pathogens in children with pneumonia. Influenza Other Respir Viruses. (2024) 18(8):e13362. 10.1111/irv.13362 39118486 PMC11310556

[6] Khales P Razizadeh MH Ghorbani S Moattari A Saadati H Tavakoli A. Prevalence of respiratory viruses in children with respiratory tract infections during the COVID-19 pandemic era: a systematic review and meta-analysis. BMC Pulm Med. (2025) 25(1):135. 10.1186/s12890-025-03587-z 40133851 PMC11934662

[7] Li T Long J Li Z Xiong Y Feng L Jiang M Epidemiology of human respiratory tract infection in Chongqing, China after COVID-19-based on surveillance data encompassing 17 respiratory pathogens. Front Cell Infect Microbiol. (2025) 15:1567341. 10.3389/fcimb.2025.1567341 40625827 PMC12229849

[8] Cilloniz C Dela Cruz C Curioso WH Vidal CH, Pneumo-Strategy. World pneumonia day 2023: the rising global threat of pneumonia and what we must do about it. Eur Respir J. (2023) 62(5):2301672. 10.1183/13993003.01672-2023 37945031

[9] Miller S Chiu C. The role of metagenomics and next-generation sequencing in infectious disease diagnosis. Clin Chem. (2021) 68(1):115–24. 10.1093/clinchem/hvab173 34969106

[10] Ruan Z Shi H Chang L Zhang J Fu M Li R The diagnostic efficacy of metagenomic next-generation sequencing (mNGS) in pathogen identification of pediatric pneumonia using bronchoalveolar lavage fluid (BALF): a systematic review and meta-analysis. Microb Pathog. (2025) 203:107492. 10.1016/j.micpath.2025.107492 40113108

[11] Yin Y Zhu P Guo Y Li Y Chen H Liu J Enhancing lower respiratory tract infection diagnosis: implementation and clinical assessment of multiplex PCR-based and hybrid capture-based targeted next-generation sequencing. EBioMed. (2024) 107:105307. 10.1016/j.ebiom.2024.105307 PMC11403251 39226681

[12] Gu D Liu J Wang J Yi Y Chu Y Gao R Integrating DNA and RNA sequencing for enhanced pathogen detection in respiratory infections. J Transl Med. (2025) 23(1):325. 10.1186/s12967-025-06342-4 40087699 PMC11907987

[13] Yang J Wang Y Yang L Wu J. Laboratory validation of targeted next-generation sequencing assay for pathogen detection in lower respiratory infection. Microbiol Spectr. (2025) 13(7):e0175124. 10.1128/spectrum.01751-24 40396715 PMC12210981

[14] Wei M Mao S Li S Gu K Gu D Bai S Comparing the diagnostic value of targeted with metagenomic next-generation sequencing in immunocompromised patients with lower respiratory tract infection. Ann Clin Microbiol Antimicrob. (2024) 23(1):88. 10.1186/s12941-024-00749-5 39350160 PMC11443791

[15] Subspecialty Group of Respiratory Diseases, The Society of Pediatrics, Chinese Medical Association, Editorial Board, Chinese Journal of Pediatrics. Guidelines for management of community acquired pneumonia in children (the revised edition of 2013) (I). Zhonghua Er Ke Za Zhi. (2013) 51(10):745–52. 10.3760/cma.j.issn.0578-1310.2013.10.006 24406226

[16] Deng Z Li C Wang Y Wu F Liang C Deng W Targeted next-generation sequencing for pulmonary infection diagnosis in patients unsuitable for bronchoalveolar lavage. Front Med (Lausanne). (2023) 10:1321515. 10.3389/fmed.2023.1321515 38179267 PMC10764475

[17] Tan J Chen Y Lu J Lu J Liu G Mo L Pathogen distribution and infection patterns in pediatric severe pneumonia: a targeted next-generation sequencing study. Clin Chim Acta. (2025) 565:119985. 10.1016/j.cca.2024.119985 39362455

[18] Mazur NI Caballero MT Nunes MC. Severe respiratory syncytial virus infection in children: burden, management, and emerging therapies. Lancet. (2024) 404(10458):1143–56. 10.1016/S0140-6736(24)01716-1 39265587

[19] Esneau C Duff AC Bartlett NW. Understanding rhinovirus circulation and impact on illness. Viruses. (2022) 14(1):141. 10.3390/v14010141 35062345 PMC8778310

[20] Tran XD Hoang VT Goumballa N Vu TN Tran TK Pham TD Viral and bacterial microorganisms in Vietnamese children with severe and non-severe pneumonia. Sci Rep. (2024) 14(1):120. 10.1038/s41598-023-50657-5 38167637 PMC10761988

[21] Groves IJ Jackson SE Poole EL Nachshon A Rozman B Schwartz M Bromodomain proteins regulate human cytomegalovirus latency and reactivation allowing epigenetic therapeutic intervention. Proc Natl Acad Sci U S A. (2021) 118(9):e2023025118. 10.1073/pnas.2023025118 33619107 PMC7936348

[22] De Groof TWM Elder EG Lim EY Heukers R Bergkamp ND Groves IJ Targeting the latent human cytomegalovirus reservoir for T-cell-mediated killing with virus-specific nanobodies. Nat Commun. (2021) 12(1):4436. 10.1038/s41467-021-24608-5 34290252 PMC8295288

[23] Jung J Seo E Yoo RN Sung H Lee J. Clinical significance of viral-bacterial codetection among young children with respiratory tract infections: findings of RSV, influenza, adenoviral infections. Medicine (Baltimore). (2020) 99(2):e18504. 10.1097/MD.0000000000018504 31914021 PMC6959858

[24] GBD 2016 Lower Respiratory Infections Collaborators. Estimates of the global, regional, and national morbidity, mortality, and aetiologies of lower respiratory infections in 195 countries, 1990-2016: a systematic analysis for the global burden of disease study 2016. Lancet Infect Dis. (2018) 18(11):1191–210. 10.1016/S1473-3099(18)30310-4 30243584 PMC6202443

[25] Liu Y Wang R Yuan Y Zhao C Wang Q Wang Y Comparison of targeted next-generation sequencing and traditional microbial culture in the diagnosis of pulmonary infections. Diagn Microbiol Infect Dis. (2024) 110(4):116534. 10.1016/j.diagmicrobio.2024.116534 39276718

[26] ESGMAC MAPS study group. Global spatiotemporal dynamics of Mycoplasma pneumoniae re-emergence after COVID-19 pandemic restrictions: an epidemiological and transmission modelling study. Lancet Microbe. (2025) 6(4):101019. 10.1016/j.lanmic.2024.101019 40024259

[27] Shah SS Test M Sheffler-Collins S Weiss AK Hall M. Macrolide therapy and outcomes in a multicenter cohort of children hospitalized with Mycoplasma pneumoniae pneumonia. J Hosp Med. (2012) 7(4):311–7. 10.1002/jhm.1904 22271440

[28] Wang YS Zhou YL Bai GN Li SX Xu D Chen LN Expert consensus on the diagnosis and treatment of macrolide-resistant Mycoplasma pneumoniae pneumonia in children. World J Pediatr. (2024) 20(9):901–14. 10.1007/s12519-024-00831-0 39143259 PMC11422262

[29] Qi R Yang Q Li H Wan Z Ruan S. Pulmonary tuberculosis versus Mycoplasma pneumoniae pneumonia in children: a retrospective analysis of clinical and imaging characteristics. Medicine (Baltimore). (2025) 104(46):e45990. 10.1097/MD.0000000000045990 41239654 PMC12622648

[30] Oishi T Narita M Matsui K Shirai T Matsuo M Negishi J Clinical implications of interleukin-18 levels in pediatric patients with Mycoplasma pneumoniae pneumonia. J Infect Chemother. (2011) 17(6):803–6. 10.1007/s10156-011-0265-7 21681500

[31] Lee KL Lee CM Yang TL Yen TY Chang LY Chen JM Severe Mycoplasma pneumoniae pneumonia requiring intensive care in children, 2010–2019. J Formos Med Assoc. (2021) 120(1 Pt 1):281–91. 10.1016/j.jfma.2020.08.018 32948415

[32] Yang S Lu S Guo Y Luan W Liu J Wang L. A comparative study of general and severe mycoplasma pneumoniae pneumonia in children. BMC Infect Dis. (2024) 24(1):449. 10.1186/s12879-024-09340-x 38671341 PMC11046970

[33] Chen J Qi X Yin Y Zhang L Zhang J Yuan S. Effects of minocycline on macrolide-unresponsive Mycoplasma pneumoniae pneumonia in children: a single-center retrospective study. Transl Pediatr. (2021) 10(11):2997–3004. 10.21037/tp-21-356 34976765 PMC8649588

[34] Centrone F Accogli M Melilli R Marziani A Orlando VA Casulli D Prevalence of macrolide-resistant Mycoplasma pneumoniae infections after the COVID-19 pandemic in southern Italy, 2023−2025. Infect Dis Ther. (2025) 14(12):2733–41. 10.1007/s40121-025-01244-w 41057669 PMC12602751

[35] Ching PR Pedersen LL. Severe pneumonia. Med Clin North Am. (2025) 109(3):705–20. 10.1016/j.mcna.2024.12.011 40185557

[36] Pickens CI Gao CA Bodner J Walter JM Kruser JM Donnelly HK An adjudication protocol for severe pneumonia. Open Forum Infect Dis. (2023) 10(7):ofad336. 10.1093/ofid/ofad336 37520413 PMC10372865

[37] Wei D Zhang L Jin F Liu F. Impact of early myocardial injury on patients with severe pneumonia. Intern Emerg Med. (2024) 19(8):2223–32. 10.1007/s11739-024-03743-z 39127867

[38] Shi H Wang T Zhao Z Norback D Wang X Li Y Prevalence, risk factors, impact and management of pneumonia among preschool children in Chinese seven cities: a cross-sectional study with interrupted time series analysis. BMC Med. (2023) 21(1):227. 10.1186/s12916-023-02951-2 37365601 PMC10294363

