# Supplementary material for: Targeted next-generation sequencing-based pathogens detection in children with severe pneumonia in the pediatric intensive care unit
Source: Front Pediatr. 2026 Apr 28;14:1825458. doi: 10.3389/fped.2026.1825458 (PMC13161149; doi:10.3389/fped.2026.1825458)
Supplement: Supplementary file 1 [file Datasheet1.pdf]

Supplementary Material

Supplementary Table 1. Comprehensive list of 225 pathogens (including 91 bacteria, 81 viruses, 43 fungi, and 10 other pathogens) and resistance genes/sites detected by tNGS.

Category	Pathogens
**Gram-positive Bacteria**	Streptococcus pneumoniae, Mycobacterium chelonae-abscessus complex, Mycobacterium simiae, Nocardia otitidiscaviarum, Streptococcus pyogenes, Mycobacterium chelonae, Mycobacterium shigaense, Nocardia tenerifensis, Streptococcus agalactiae, Mycobacterium abscessus, Mycobacterium xenopi, Nocardia africana, Streptococcus mitis group, Mycobacterium abscessus subsp. bolletii, Mycobacterium fortuitum, Nocardia nova, Streptococcus anginosus group, Mycobacterium abscessus subsp. massiliense, Mycobacterium smegmatis, Nocardia transvalensis, Streptococcus intermedius, Mycobacterium abscessus subsp. abscessus, Nocardia spp., Corynebacterium diphtheriae, Staphylococcus aureus, Mycobacterium asiaticum, Nocardia farcinica, Trueperella pyogenes, Staphylococcus lugdunensis, Mycobacterium celatum, Nocardia cyriacigeorgici, Corynebacterium striatum, Mycobacterium tuberculosis complex, Mycobacterium gordonae, Nocardia brasiliensis, Rhodococcus equi, Nontuberculous mycobacteria, Mycobacterium kansasii, Nocardia abscessus, Parvimonas micra, Mycobacterium avium complex, Mycobacterium malmoense, Nocardia asteroides, Tropheryma whipplei, Mycobacterium avium, Mycobacterium scrofulaceum, Nocardia caviae, Listeria monocytogenes, Mycobacterium intracellulare, Mycobacterium schima
**Gram-negative Bacteria**	Klebsiella pneumoniae, Bordetella parapertussis, Klebsiella aerogenes, Moraxella catarrhalis, Klebsiella variicola, Pseudomonas aeruginosa, Bordetella pertussis, Bordetella holmesii, Klebsiella oxytoca, Escherichia coli, Pasteurella multocida, Serratia marcescens, Proteus mirabilis, Fusobacterium necrophorum, Fusobacterium nucleatum, Bacteroides fragilis, Haemophilus influenzae, Brucella spp, Legionella spp, Legionella pneumophila, Legionella bozemanii, Legionella longbeachae, Legionella maceachernii, Elizabethkingia spp., Elizabethkingia anophelis, Elizabethkingia meningoseptica, Stenotrophomonas maltophilia, Citrobacter freundii complex, Enterobacter cloacae complex, Acinetobacter calcoaceticus-baumannii complex, Acinetobacter baumannii, Acinetobacter junii, Burkholderia cepacia complex, Burkholderia cenocepacia, Burkholderia mallei, Acinetobacter ursingii, Burkholderia cepacia, Burkholderia contaminans, Burkholderia multivorans, Neisseria meningitidis, Burkholderia pseudomallei
**DNA Viruses**	Herpes simplex virus 1 (HSV-1), Herpes simplex virus 2 (HSV-2), Varicella-zoster virus (VZV), Epstein-Barr virus (EBV), Cytomegalovirus (CMV), Human herpesvirus 6 (HHV-6), Human

	herpesvirus 6A (HHV-6A), Human herpesvirus 6B (HHV-6B), Human herpesvirus 7 (HHV-7), Human adenovirus, Human adenovirus species B, Human adenovirus type 3, Human adenovirus type 7, Human adenovirus type 11, Human adenovirus type 14, Human adenovirus type 21, Human adenovirus type 34, Human adenovirus type 35, Human adenovirus type 55, Human adenovirus species C, Human adenovirus type 1, Human adenovirus type 2, Human adenovirus type 5, Human adenovirus type 6, Human adenovirus type 57, Human adenovirus group D, Human adenovirus type 4, Human bocavirus, Human bocavirus 1, Human bocavirus 2, Human bocavirus 3, Human bocavirus 4, BK polyomavirus (BKPyV), Human parvovirus B19, WU polyomavirus (WUPyV), JC polyomavirus (JCPyV)
**RNA Viruses**	Human respiratory syncytial virus A, Human respiratory syncytial virus B, SARS-CoV-2, Human coronavirus 229E, Human coronavirus HKU1, Human coronavirus NL63, Human coronavirus OC43, Human parainfluenza virus, Human respirovirus 1 (Human parainfluenza virus 1), Human rubulavirus 2 (Human parainfluenza virus 2), Human respirovirus 3 (Human parainfluenza virus 3), Human rubulavirus 4 (Human parainfluenza virus 4), Influenza A virus, Influenza A virus H1N1(2009), Influenza A virus H1N1, Influenza A virus H7N9, Influenza A virus H3N2, Influenza A virus H5N1, Influenza B virus Victoria lineage, Influenza B virus, Influenza C virus, Influenza B virus Yamagata lineage, Rhinovirus type A, Enterovirus, Coxsackievirus A10, Rhinovirus, Rhinovirus B, Coxsackievirus A2, Enterovirus A, Coxsackievirus A5, Enterovirus group B, Coxsackievirus B3, Echovirus E18, Enterovirus C, Measles virus, Enterovirus D, Enterovirus D68, Human metapneumovirus, Rubella virus, Mumps virus, Enterovirus A71, Coxsackievirus A6, Rhinovirus C, Coxsackievirus A16, Echovirus E30,
**Fungi**	Aspergillus spp., Aspergillus niger complex, Aspergillus fumigatus, Aspergillus terreus complex, Aspergillus flavus complex, Cryptococcus spp., Cryptococcus gattii, Cryptococcus neoformans, Cryptococcus laurentii, Pichia kudriavzevii (Candida krusei), Rhizopus spp., Rhizopus delemar, Rhizopus microsporus, Rhizopus oryzae, Rhizomucor spp., Rhizomucor pusillus, Mucor spp., Mucor irregularis, Mucor racemosus, Lichtheimia spp., Lichtheimia corymbifera, Lichtheimia ramosa, Scedosporium spp., Scedosporium apiospermum, Scedosporium boydii, Syncephalastrum spp., Cunninghamella spp., Microascus spp., Coccidioides spp., Talaromyces marneffei, Pneumocystis jirovecii, Trichosporon asahii, Arthrinium phaeospermum, Histoplasma capsulatum, Fusarium spp., Candida spp., Candida albicans, Candida parapsilosis, Candida metapsilosis, Candida tropicalis, Candida auris, Candida glabrata, Meyerozyma guilliermondii
**Mycoplasma, Chlamydia,**	Mycoplasma pneumoniae, Ureaplasma urealyticum, Ureaplasma parvum, Mycoplasma hominis, Chlamydophila pneumoniae, Chlamydia

**Rickettsia, etc.**	trachomatis, Chlamydophila psittaci, Coxiella burnetii, Leptospira spp.
**Parasite**	Paragonimus westermani

**Drug resistance**	**Target gene/site**
**Carbapenemase genes-class A**	bla_KPC, bla_SME, bla_IMI, bla_GES
**Carbapenemase genes-class B**	bla_NDM, bla_IMP, bla_VIM, bla_SPM, bla_GIM
**Carbapenemase genes-class D**	bla_OXA-48
**Methicillin-resistant staphylococcus resistance gene**	mecA
**Extended-spectrum β-lactamase (ESBLs)**	bla_CTX-M
**Mycoplasma pneumoniae 23S rRNA gene**	2063G, A2064G, A2067G, C2617G
**Bordetella pertussis 23S rRNA gene**	A2047G
**Aspergillus fumigatus cyp51A gene**	TR46, Y121F, T289A
